# Morphological and Molecular Characterization of *Paragonimus* Species Isolated from Freshwater Crabs in Southern Yunnan, China

**DOI:** 10.1155/2021/5646291

**Published:** 2021-12-31

**Authors:** Qiu-Hong Shu, Yang Yang, Miao-Miao Wang, Shu-De Li, Ming Tian, Wen-Wei Bai, Yong Meng, Shu-Mei-Qi He, Wen-Lin Wang

**Affiliations:** ^1^Department of Cardiology, The Second Affiliated Hospital of Kunming Medical University, No. 374, Dianmian Road, Kunming 651010, Yunnan, China; ^2^Oncology Department, People's Hospital of Xishuangbanna Dai Autonomous Prefecture, Xishuangbanna 666100, Yunnan, China; ^3^School of Basic Medical Sciences, Kunming Medical University, No. 1168, Chunrong West Road, Yuhua Street, Chenggong District, Kunming 65050, Yunnan, China

## Abstract

*Paragonimus* species are highly prevalent in various regions of China. The study's objective is to isolate and identify *Paragonimus* from natural habitats and compare the phylogenetic diversity of *Paragonimus* in southern Yunnan province, China. Metacercariae of *Paragonimus* was isolated from crabs, and morphologic identification was performed by microscopy. Metacercariae were injected into experimental *Paragonimus* free Sprague Dawley rats. After 114 days, adult worms and eggs were isolated from multiple organs. Morphologic identification confirmed the initial identification. DNA was extracted from 5 adult worms, and molecular characterization was performed by amplification and sequencing of CO1 and ITS2 regions, followed by phylogenetic analysis. Out of 447 crabs captured, 186 crabs were found to be infected. A total of 4 species of *Paragonimus* was observed from naturally infected crabs. *Paragonimus microrchis* (2), *Paragonimus heterotremus* (1), *Paragonimus proliferus* (1), and *Paragonimus skrjabini* (1) were isolated and identified. A total of 32 sequences were downloaded from the National Center for Biotechnology Information, and 5 sequences generated in the study were used for phylogenetic analysis. In the phylogenetic tree of the CO1 gene, *Paragonimus proliferus*, *Paragonimus heterotremus*, and *Paragonimus skrjabini* were clustered with the same species, and the confidence values of their branches were >95%. A congruent phylogenetic relationship was observed with the ITS2 phylogenetic tree. In the phylogenetic tree constructed with the combined dataset of CO1 and ITS2 datasets, *Paragonimus proliferus*, *Paragonimus heterotremus*, and *Paragonimus skrjabini* clustered with the same species, and their branch confidence values were >94%. *Paragonimus microrchis* clustered with *Paragonimus bangkokensis* in both datasets. Phylogenetic analysis revealed robustness of the double loci method as against the single-locus method with either CO1 or ITS2 alone. *Paragonimus* species isolated from the southern Yunnan province, China, was phylogenetically diverse, and the analysis revealed the clustering of multiple species of *Paragonimus* isolated from different geographic locations.

## 1. Introduction

Trematodes belonging to the genus *Paragonimus* are important parasites causing zoonotic infections in vertebrates, including human beings. They are of socioeconomic importance since the route of infection is mainly food-borne [1]. The life cycle of *Paragonimus* is relatively complex that requires a minimum of three hosts, including a definitive host and two intermediate hosts [2]. The first and second intermediate hosts are frequently snails belonging to the families Assimineidae and Hydrobiidae and crabs belonging to the families Potamidae and Parathelphusidae [2]. Different species of *Paragonimus* have their own predilection for infecting specific genera of snails and crabs and hence the epidemiological prevalence of different species of *Paragonimus* is determined by the existence of suitable hosts. Some species of *Paragonimus* have been identified only from intermediate hosts in certain geographies which suggests infection and maintenance in nonhuman mammals [3, 4].

The genus *Paragonimus* is rich in species diversity that has been reported from varied geographies, including Asia, Africa, and Central and South America spanning both tropical and temperate climates [5]. The species diversity is also reflected by the various phylogenies of *Paragonimus* and mainly comprises 3 species complexes. *Paragonimus westermani* is the most commonly reported species complex found in South and South East Asia [6]. *Paragonimus heterotremus* species has been predominantly isolated and reported from South East Asia while the *Paragonimus skrjabini* complex has been reported from China and East Asia [7, 8]. Usually, the adult worms are large, plump, and resemble a coffee bean in size and shape. The most common discrimination is the patterns of lobation of the ovary and testes. For instance, the ovary of *Paragonimus ohirai* and *Paragonimus mexicanus* possesses many delicate branches, while that of *Paragonimus westermani* has 6 simple lobes [9, 10]. The allelic diversity and ontogenetic changes have been previously studied with isozymes, which have been superseded with molecular approaches [5]. There were previous instances of discordance in morphology and the ontogenetic changes predicted by both molecular and isozyme-based methods [11]. One of the main reasons for the observed discordance is the lack of clarity on the species boundaries and also mendelian polymorphism leading to morphological changes causing the creation of distinct species that are interfertile [12].

DNA sequence data can be used to construct phylogenetic trees that can be used to infer evolutionary trends, including insights into speciation and geographic spread [13, 14]. Further, the morphological differences in adult and metacercariae that could be used to differentiate different species are also limited. This expands the potential role of DNA sequence analysis to assist in confirming species distribution and phylogenetic diversity. Currently, nuclear (internal transcribed spacer 2 (ITS2) and mitochondrial DNA (cytochrome c oxidase subunit 1 (CO1)) sequences are used for assessing the phylogeographies in various organisms [15, 16].

Over 50 species of the *Paragonimus* have been reported worldwide, of which China has the largest distribution and number of species [17]. Due to the migration of people from rural to urban areas and food eating habits such as continuous consumption of raw or undercooked freshwater crab or meat such as wild boar or venison [2, 18], China represents an ecological hotspot for the dissemination and further evolution of *Paragonimus* species. Moreover, the diagnosis of paragonimiasis is difficult due to nonspecific clinical symptoms [18]. Hence, frequent phylogenetic analysis of *Paragonimus* species from nonmammalian hosts will help understand the genetic variation and also suggest suitable lifestyle modifications for populations at risk of acquiring paragonimiasis. Yunnan province has the most endemic species of *Paragonimus* due to its mountainous plateau topography and unique geographical environment [17]. Hence, in this study, we report the phylogenetic diversity and evolutionary relationship of *Paragonimus* species isolated from crabs in southern Yunnan province, China.

## 2. Materials and Methods

### 2.1. Parasitological Methods

The *Paragonimus* metacercariae were isolated from naturally infected primary freshwater crabs belonging to the genus *Indochinamon*, the second intermediate hosts, from Tongchang Town, Jinping County, Yunnan Province, China. The habitat included fast-moving streams with no adjacent vegetation. The identification of the secondary hosts was done according to the classification method of “Chinese Medical Crustaceans” [19]. The crabs were smashed in a mortar followed by sieving and washed with distilled water into a sedimentation cup. The debris was passed through the first filter mesh of pore size 200 microns, and later the filtered liquid and sediment were passed through a filter pore size of 1000 microns to collect the sediment. The supernatant obtained during this process was discarded after every 20 minutes, and the same step was repeated four to five times until the supernatant was clear. The sediment obtained at the bottom of the cup was then placed in a glass dish for microscopic biological observation. The metacercariae of *Paragonimus* were counted under the microscope and a part of the sediment with the metacercariae were fixed with 70% absolute ethanol and stored in a refrigerator at 4°C with 96% ethanol for molecular biological experiments.

### 2.2. Experimental Infection

Freshly isolated, live metacercariae were then injected intraperitoneally (15 metacercariae per rat) into paragonimiasis-negative Sprague Dawley (SD) rats (purchased from the Laboratory Animal Department of Kunming Medical University). All the animals were handled in accordance with the Guide for the Care and Use of Laboratory Animals published by the US National Institutes of Health (NIH Publication No. 8523, revised 1985). All experimental protocols were approved by the Animal Care and Use Committee of Kunming Medical University (reference: KMMU2015002). Experimental animal inoculation was performed based on the sampling point. After subcutaneous injection, five SD rats were sacrificed on the second, fourth, and sixth week to confirm infection of rats with cercariae of *Paragonimus*. After 114 days of injection, the SD rats were dissected to isolate the cysts, eggs, and adult worms from the muscles, abdomen, liver, thoracic cavity, and lungs of SD rats. The isolated adult worms were used for genomic DNA extraction and for preparing permanent slides for microscopic confirmation by fixing them onto glass slides with alcohol, formalin, and acetic acid.

### 2.3. Microscopic Identification

The different characteristic features such as the shape, body size, measurement, and position of the suckers were used for microscopic and morphologic characterization.

### 2.4. Molecular Analysis

Genomic DNA was extracted from both the adult worms in SD rats and the metacercariae extracted from crab using QIAamp DNA Mini kit (QIAGEN, Hilden, Germany). The whole process was carried out in strict accordance with the manufacturer's instructions. The final elution of DNA was done with 100 *µ*L of distilled water. The extracted total genomic DNA was quantified and stored in the refrigerator at−20°C until further use.

### 2.5. Polymerase Chain Reaction Amplification of CO1 and ITS2

Polymerase chain reaction (PCR) was performed with primers targeting a fragment of the CO1 gene and the ITS2 region synthesized by Shanghai Bioengineering Co., Ltd. The primers used for amplifying CO1 gene fragments were CO1F-5′GAGGTGTATGTCCTGATTTTGCC-3′ and CO1R-5′GACCTCACCCAATGACCCTGCAACA3′, and the primers for amplifying ITS2 gene fragments were ITS2F-5′GGGTACCGGATCACTCGCTCGGTG3′ and ITS2R-5′GGGGATCCTGGTTGCCTTAGTCTCCGC3′ [20]. PCR was performed in 25 *µ*L volume with 2 *µ*L template DNA corresponding to 0.1 ng and 1 *µ*L of primers (10 *µ*mol/*µ*L), 2.5 *µ*L of 10X PCR buffer, 1 *µ*L of 10 mM deoxynucleotide triphosphates (dNTP), 0.1 *µ*L (0.5 units) of Taq enzyme (5 U/ul), and 17.4 *µ*L of PCR grade water. The setup of PCR was done in an ice bath. The PCR amplification was conducted in TaKaRa PCR instrument (Baolingbao Biology Co., Ltd., China), and the amplification conditions were as follows: initial denaturation of 95°C for 3 minutes followed by 35 cycles of denaturation at 93°C for 1 minute, annealing at 48°C (for CO1)/60°C (for ITS2) for 1 minute, and extension at 72°C for 1 minute followed by a final extension at 72°C for 5 minutes. The expected length of the PCR fragments was 500–750 base pairs. Detection of PCR amplified products was done by agarose gel electrophoresis with 1.5% agarose gel immersed in 1.0% Tris-Acetate-EDTA buffer stained with ethidium bromide. The purity and quantity were estimated by imaging the gel in a gel documentation system (Bio-Rad company).

The PCR products were then subjected to bidirectional sequencing using the same PCR primers by Shanghai Biotechnology Co., Ltd. (Hitachi fluorescent DNA sequencer SQ-3000). The forward and reverse sequences were then manually curated and aligned with the Dnastar v7.1 software, and the consensus sequence was used for bioinformatic analysis.

The initial quality check of the sequences was done by checking the coverage and alignment with previously submitted sequences in National Center for Biotechnology Information (NCBI) using the BLAST tool. Previously submitted sequences of CO1 and ITS2 were retrieved from NCBI and compared with the sequences obtained in this study with ClustalX software with default parameters. Phylogenetic analysis was done as per the Kimura 2-parameter model. The neighbor-joining method (NJ) and maximum parsimony (MP) method were used to construct the phylogenetic tree. The genetic distances of the Kimura-2-parameter model were calculated, considering all substitutions and missing/gaps as unambiguous changes. Test of phylogenetic accuracy was done with bootstrap replicates of 1000. The analysis was performed with MEGA5.0 software. The cut-off value for the consensus tree was set to 75%.

## 3. Results

### 3.1. Morphological Identification

#### 3.1.1. Metacercariae

A total of 447 crabs captured from the stream in Tongchang Town, Jinping County, Yunnan Province, China, were included for this study. Out of 447 crabs, 186 crabs were found to be infected. A total of 551 metacercariae were isolated and identified from the 186 crabs (2.96 per crab). The size of the metacercariae was found to be 0.419 mm ∗ 0.398 mm. The average thicknesses of the outer wall and inner wall were found to be 0.004 mm and 0.012 mm, respectively, using the microscopic technique. Further, the larvae were surrounded by two larger excretory cysts and intestinal branches.

#### 3.1.2. Adult Worm

**Figure 1:**
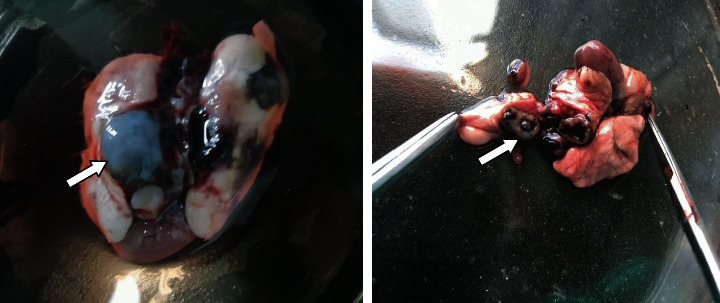
*Paragonimus* cyst extracted from Sprague Dawley rats after 112 days.

After experimental infection, from each SD rat, 2 to 4 adult worms were retrieved (Figure 1). Adult worms were distributed in both lungs and muscles but seldom found in the liver and brain of affected SD rats. A total of 5 fully developed adult flukes recovered from SD rats were used for morphological and molecular analysis. The average size of the gravid adult worms was 6.7 mm ∗ 3.8 mm. The tegmental spines were mainly clustered into clusters of 4–6 around the ventral sucker. The spines around the oral sucker were short, small, and solitary. The size of the oral and ventral suckers was 0.43 mm ∗ 0.53 mm and 0.75 mm ∗ 0.79 mm, respectively. The reproductive organs were observed as large uterine masses, with a size of 2.695 mm ∗ 2.107 mm. The testicle is in the inferior half of the worm with dimensions of 1.72 mm ∗ 1.38 mm.

Morphological identification of the metacercariae isolated from the infected crabs and adult worms from the SD rats led to the identification of 4 species of *Paragonimus*: *Paragonimus proliferus*, *Paragonimus microrchis*, *Paragonimus heterotremus*, and *Paragonimus skrjabini*.

### 3.2. Molecular Identification

A total of 5 samples (all adult worms) of *Paragonimus* from southern Yunnan province were used for molecular phylogenetic analyses. The sequences were aligned with those of several related species within genus *Paragonimus* obtained from GenBank. PCR amplification of the CO1 and ITS2 regions, followed by agarose gel electrophoresis, revealed amplicons of about 500 and 750 bp in length, respectively. BLAST searches using our sequences as queries found matches with 100% coverage in GenBank. Further, only the CO1 sequence of *Paragonimus microrchis* differed slightly from previously published sequences. All the other sequences obtained in this study revealed 100% identity to previously published sequences.

#### 3.2.1. Base Composition of DNA Sequence in *Paragonimus* Species

The base composition ratio of the same gene in different species was identical. However, the ratios of DNA base composition between CO1 and ITS2 among different isolates were different. In the CO1 gene, the content of base *T* was very high, with an average of 43.2%, far exceeding the base composition of ITS2, which is consistent with the abundant base *A* and base *T* content of the mitochondrial genome (Table 1).

#### 3.2.2. Genetic Distance between ITS2 and CO1 of *Paragonimus* Species

**Figure 2:**
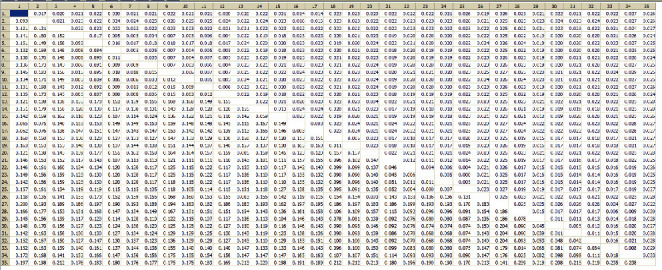
The genetic distance matrix of CO1 of *Paragonimus* species under the K-2 parameter model. *Note*. In the table, the lower triangle is the genetic distance, and the upper triangle is the standard error. The samples corresponding to the numbers in the sidebar are as follows: (1) *P. bangkokensis* AB354227 Thailand; (2)*P. bangkokensis* AB735645 Vietnam; (3) *P. harinasutai* AB354604 Zhejiang-China; (4) *P. heterotremus* AB270676 Vietnam; (5) *P. heterotremus* AB325517 India; (6) *P. heterotremus* AB354229 Thailand; (7) *P. heterotremus* AB827370 Vietnam; (8) *P. heterotremus* HM627190 Yunnan-China; (9) *P. heterotremus* KC859926 Myanmar; (10) *P. heterotremus* KC859927 Thailand; (11) *P. heterotremus* KC859933 Thailand; (12) *P. heterotremus* Sample 29; (13) *P. iloktsuenensis* AF008188; (14) *P. macrorchis* AF159598 Thailand; (15) *P. macrorchis* KP784350 Laos; (16) *P. microrchis* Sample 31; (170 *P. microrchis* Sample 32; (18) *P. miyazakii* AY618807 Japan; (19) *P. miyazakii* AY618834 Fujian-China; (20) *P. ohirai* U97214 Japan; (21) *P. proliferus* AB663681 Vietnam; (22) *P. proliferus* EU401809 Yunnan-China; (23) *P. proliferus* EU401811 Yunnan-China; (24) *P. proliferus* EU401812 Yunnan-China; (25) *P. proliferus* Sample 30; *(2*6) *P. sadoensis* AF008190; (27) *P. siamensis* JQ322632 India; (28) *P. skrjabini* AB703456 Vietnam; (29) *P. skrjabini* AY618759 Guangxi-China; (30) *P. skrjabini* AY618760 Sichuan-China; (31) *P. skrjabini* AY618763 Hubei-China; (32) *P. skrjabini* AY618801 Guangdong-China; (33) *P. skrjabini* AY618805 Yunnan-China; (34) *P. skrjabini* Sample 28; (35) *P. westermani* AB354223 Thailand.

**Figure 3:**
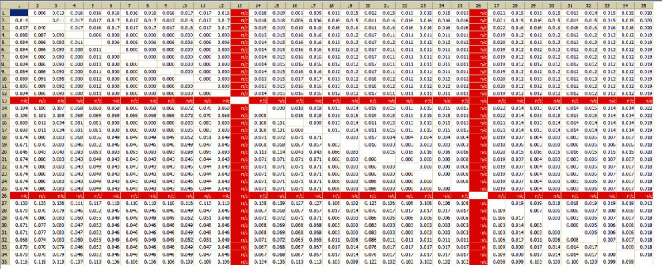
The genetic distance matrix of ITS2 of Paragonimus species under the K-2 parameter model. *Note*. In the table, the lower triangle is the genetic distance, and the upper triangle is the standard error. The ITS2 data of *P. iloktsuenensis* and *P. sadoensis* are missing, and the genetic distance to the ITS2 of other paragonimites is invalid using n/c. The samples corresponding to the numbers in the sidebar are as follows: (1) *P. bangkokensis* AB354227 Thailand; (2) *P. bangkokensis* AB735645 Vietnam; (3) *P. harinasutai* AB354604 Zhejiang-China; (4) *P. heterotremus* AB270676 Vietnam; (5) *P. heterotremus* AB325517 India; *(6) P. heterotremus* AB354229 Thailand; (7) *P. heterotremus* AB827370 Vietnam; (8) *P. heterotremus* HM627190 Yunnan-China; (9) *P. heterotremus* KC859926 Myanmar; *(10) P. heterotremus* KC859927 Thailand; (11) *P. heterotremus* KC859933 Thailand; (12) *P. heterotremus* Sample 29; (13) *P. iloktsuenensis* AF008188; (14) *P. macrorchis* AF159598 Thailand; (15) *P.* macrorchis KP784350 Laos; (16) *P. microrchis* Sample 31; (17) *P. microrchis* Sample 32; (18) *P. miyazakii* AY618807 Japan; (19) *P. miyazakii* AY618834 Fujian-China; (20) *P. ohirai* U97214 Japan; (21) *P. proliferus* AB663681 Vietnam; (22) *P. proliferus* EU401809 Yunnan-China; (23) *P. proliferus* EU401811 Yunnan-China; (24) *P. proliferus* EU401812 Yunnan-China; (25) *P. proliferus* Sample 30; (26) *P. sadoensis* AF008190; (27) *P. siamensis* JQ322632 India; (28) *P. skrjabini* AB703456 Vietnam; (29) *P. skrjabini* AY618759 Guangxi-China; (30) *P. skrjabini* AY618760 Sichuan-China; (31) *P. skrjabini* AY618763 Hubei-China; (32) *P. skrjabini AY618801* Guangdong-China; (33) *P. skrjabini* AY618805 Yunnan-China; (34) *P. skrjabini* Sample 28; (35) *P. westermani* AB354223 Thailand.

The average genetic distance of *Paragonimus* species for the ITS2 sequence was 0.056, while the average genetic distance for the CO1 sequence was 2.3 times ITS2 with 0.128. The genetic distances of adult worms had extremely high sequence similarity though the distances between DNA and the corresponding species were the smallest (Figures 2 and 3).

#### 3.2.3. Phylogenetic Analysis

**Figure 4:**
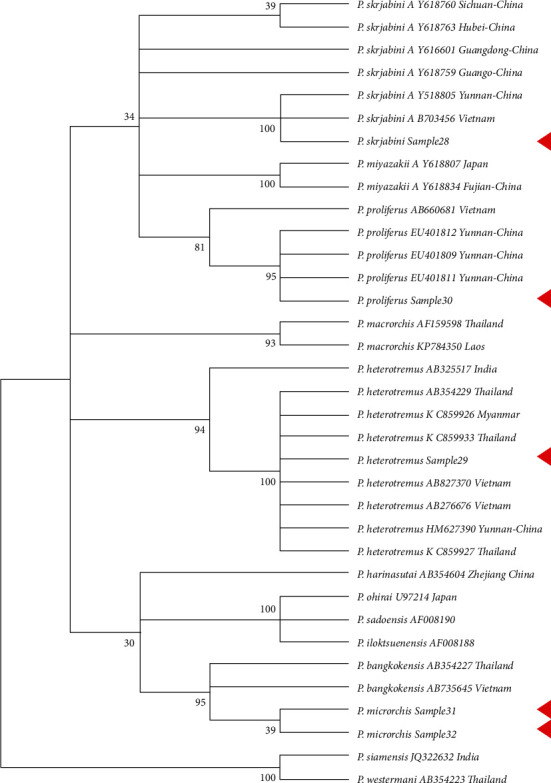
Phylogenetic tree of *Paragonimus* CO1 gene adjacency (NJ). Samples 28, 29, 30, 31, and 32 were sequences obtained in this study.

**Figure 5:**
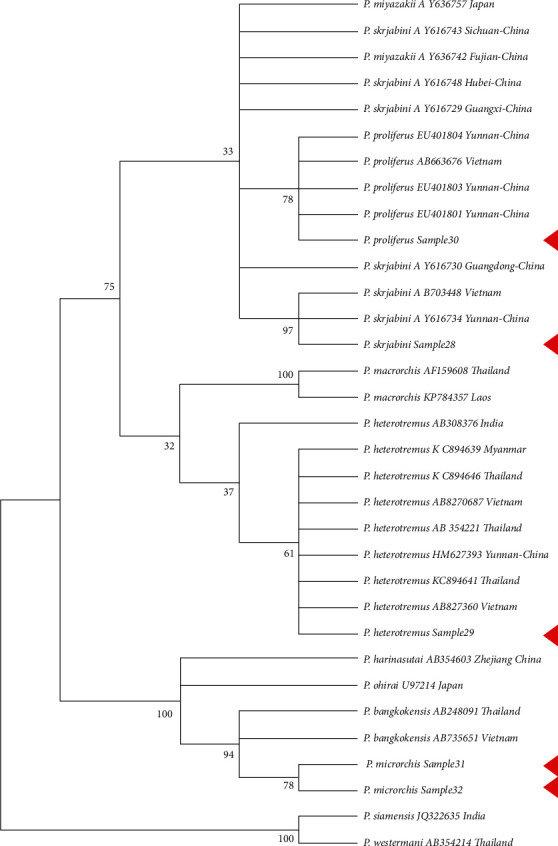
Maximum simplified phylogenetic tree (MP) of ITS2 DNA of *Paragonimus*. Samples 28, 29, 30, 31, and 32 were sequences obtained in this study.

**Figure 6:**
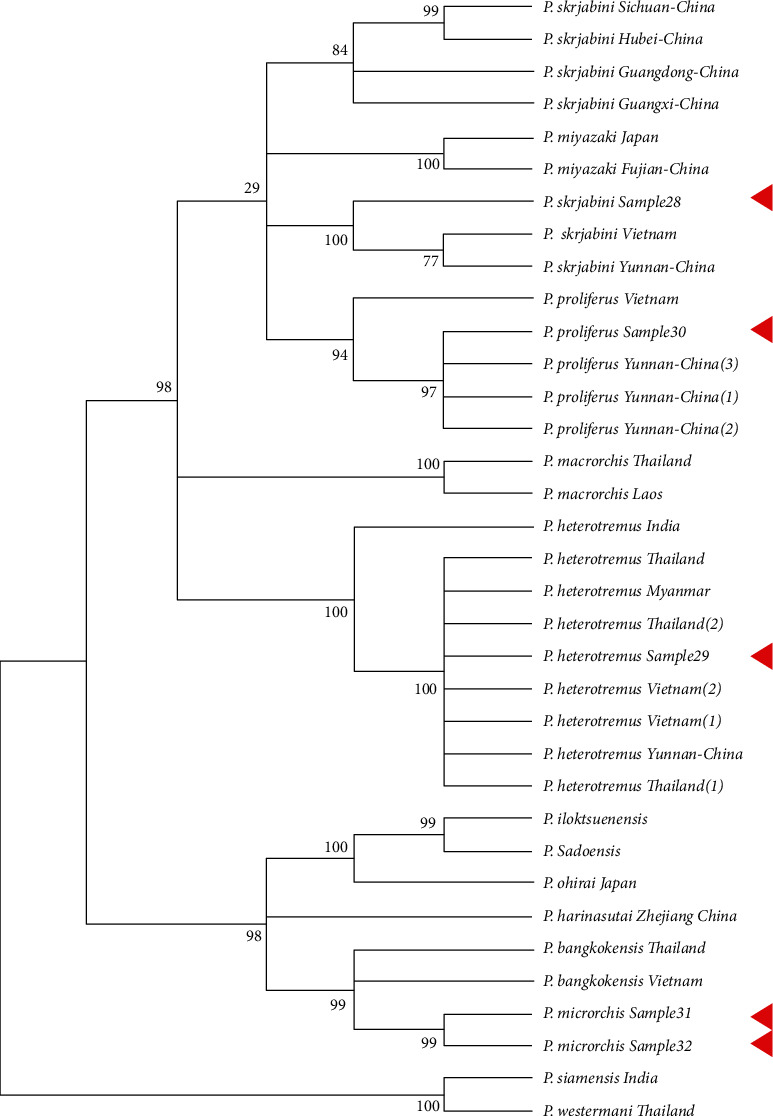
Adjacency (NJ) phylogenetic tree of the combined dataset of CO1 and ITS2 DNA of *Paragonimus* Samples 28, 29, 30, 31, and 32 were sequences obtained in this study.

A total of 30 gene sequences were downloaded from NCBI and were selected based on coverage for this analysis (Table 2). In the phylogenetic tree of the CO1 gene, *Paragonimus proliferus*, *Paragonimus heterotremus*, and *Paragonimus skrjabini* were clustered with the same species, and the confidence values of their branches were more than 95% (except for the support degree of the branches where sample 30 was located at 88%) (Figure 4). *Paragonimus microrchis* clustered with *Paragonimus bangkokensis* (Figure 5). A congruent phylogenetic relationship was observed with the ITS2 phylogenetic tree. In the phylogenetic tree constructed with the combined dataset of CO1 and ITS2 datasets, *Paragonimus proliferus*, *Paragonimus heterotremus*, and *Paragonimus skrjabini* still clustered with the same species, and their branch confidence values were more than 94%. *Paragonimus microrchis* remained clustered with *Paragonimus bangkokensi* (Figure 6).

## 4. Discussion

China is known for the endemic diversity of *Paragonimus* species, wherein 45 genera and 311 species have been identified till 2018 [21]. A previous paper reported that Yunnan alone has the highest number of species (48) and genera [14] and the second-highest Shannon index (2.21%) [22]. This number has recently increased to 58 species [23]. Hence, we determined the occurrence and phylogenetic diversity of *Paragonimus* species in southern Yunnan province, China. In the current study, out of the sequenced samples, 2 were *Paragonimus microrchis* and 1 each of *Paragonimus heterotremus*, *Paragonimus skrjabini*, and *Paragonimus proliferus.*

Since the differential identification features of cercariae are not well marked, most often, the species identification is done at the metacercariae stage from the secondary intermediate hosts [24]. In this study, morphological identification made at the metacercariae stage and adult stage were concordant with each other, further substantiating the role of morphological identification. This was also confirmed with PCR amplification and sequencing of CO1 and ITS2 region.

Phylogenetic analysis with previously published sequences and the sequences generated in this study revealed concordance between the single locus (CO1 or ITS2) and double loci (CO1 and ITS2) phylogenetic analysis. The phylogenetic analysis revealed the presence of 3 distinct clusters of *Paragonimus* consisting of *Paragonimus siemensis* and *Paragonimus westermani* in cluster 1; *Paragonimus skrjabini*, *Paragonimus miyazakii*, *Paragonimus proliferus*, *Paragonimus macrorchis*, and *Paragonimus heterotremus* in cluster 2; and *Paragonimus microrchis*, *Paragonimus bangkokensis*, *Paragonimus harinasutai*, *Paragonimus iloktsuensis*, and *Paragonimus sadoensis* in cluster 3. Extensive branching was observed in cluster 2 wherein *Paragonimus skrjabini* isolated from different regions in China showed distinct branches. Further, *Paragonimus heterotremus* clustered into a stable branch with *Paragonimus heterotremus* from South East Asia clustering into a single monophyletic branch group distinct from the *Paragonimus heterotremus* from India. Similarly, *Paragonimus proliferus* also clustered as a monophyletic group in most cases (except in NJ trees constructed by ITS2). The relationship between *Paragonimus macrorchis* and the first two branches is not clear. However, in the MP phylogenetic tree of ITS2 datasets, CO1 and ITS2 combined datasets, *Paragonimus heterotremus* tends to be clustered into one branch.

By comparing the results of constructing adjacent NJ and MP phylogenetic trees from CO1, ITS2, CO1, and ITS2 datasets, it is obvious that different datasets and tree-building methods have a great influence on the robustness of phylogenetic trees. In phylogenetic trees constructed from single-gene datasets, the number of branches with more than 75% support is less than that constructed from joint datasets, especially self-exhibition, which deserves significant improvement. The phylogenetic tree constructed by the MP method using CO1 and ITS2 datasets has high robustness, and *Paragonimus macrorchis* and *Paragonimus heterotremus* were clustered together, which is supported by 91%.

Among the *Paragonimus* spp. reported in this study, all the species were previously reported from China. A study by Lou et al. utilized similar approaches in identifying the phylogenetic diversity of *Paragonimus* in China and reported clustering of isolates based on geographic location, which was in accordance with the current study [25]. *Paragonimus skrjabini* was previously reported from Gansu, Shanxi, Yunnan, Guangxi, Guizhou, Sichuan, and Jiangxi provinces of China. In contrast, *P. heterotremus* has been reported only from Yunnan and Guangxi provinces [2].

Phylogenetic analysis has varied values in different organisms. In *Paragonimus*, where there is no consensus on identification with both morphological and molecular characteristics, phylogenetic analysis helps in determining the phylogeographies [26, 27]. The results of our study further substantiated the conservative phylogeographies revealed through analysis of Cox1 and ITS2 regions. Previous literature on the identification of *Paragonimus* species among individuals consuming freshwater crabs is scarce. A recent study reported that 8 adult humans were infected with *P. heterotremus*, which were recovered from their lung masses on examination in Xishuangbanna, Yunnan [28]. In such a case, phylogenetic analysis is helpful in the identification of various species of organisms.

The study has certain limitations. The study sites were localized to southern Yunnan province in China; hence, the results may not be extended to other parts of China. Furthermore, we used the maximum parsimony method, which is infrequently used despite the robustness mainly because of the nature of assumptions deployed in the method. Nevertheless, the maximum likelihood method used in other studies also has drawbacks.

In conclusion, we obtained 4 morphologically different species of *Paragonimus* from southern Yunnan province, China. Phylogenetic analysis using the polymorphisms in two different loci revealed considerable variations in the species of *Paragonimus* identified in different geographic locations with clustering of *Paragonimus* based on geographic location.

## Figures and Tables

**Table 1:** Base composition of *Paragonimus* species measured by CO1 and ITS2 gene fragments.

Samples	CO1					ITS2				
	T	C	A	G	Total	T	C	A	G	Total
Sample 29 (*P. heterotremus*)	45.0	10.7	16.1	28.2	350.0	28.1	24.7	16.9	30.3	327.0
Sample 31 (*P. microrchis*)	41.5	12.9	16.7	28.9	350.0	28.7	25.1	16.0	30.3	327.0
Sample 32 (*P. microrchis*)	41.3	12.4	16.8	29.5	350.0	28.7	25.1	16.0	30.3	327.0
Sample 30 (*P. proliferus*)	43.5	9.8	17.9	28.8	350.0	27.6	26.0	15.7	30.7	327.0
Sample 28 (*P. skrjabini*)	40.6	11.1	18.4	29.9	350.0	28.7	25.4	15.2	30.7	327.0
Avg.	43.2	11.1	17.3	28.3	350.0	28.1	25.4	16.1	30.4	327.0

**Table 2:** DNA sequences used for the phylogenetic analysis.

Serial number (no.)	Generic name (generic name)	Species name (specific name)	GenBank serial number (accession number)		Sampling point (location of sample)	Specimen number (sample code)^*∗*^
			CO1	ITS2		
1	*Paragonimus*	*bangkokensis*	AB354227	AB248091	Thailand (Thailand)	
2	*Paragonimus*	*bangkokensis*	AB735645	AB735651	Vietnam	
3	*Paragonimus*	*harinasutai*	AB354604	AB354603	Zhejiang-China (Zhejiang province, China)	
4	*Paragonimus*	*heterotremus*	AB270676	AB270687	Vietnam	
5	*Paragonimus*	*heterotremus*	AB325517	AB308376	India (India)	
6	*Paragonimus*	*heterotremus*	AB354229	AB354221	Thailand (Thailand)	
7	*Paragonimus*	*heterotremus*	AB827370	AB827360	Vietnam	
8	*Paragonimus*	*heterotremus*	HM627190	HM627193	Yunnan-China (Yunnan province, China)	
9	*Paragonimus*	*heterotremus*	KC859926	KC894639	Myanmar (Myanmar)	
10	*Paragonimus*	*heterotremus*	KC859927	KC894641	Thailand (Thailand)	
11	*Paragonimus*	*heterotremus*	KC859933	KC894646	Thailand (Thailand)	
12	*Paragonimus*	*heterotremus*	MN656988	—	Yunnan-China (Yunnan province, China)	Sample29
13	*Paragonimus*	*iloktsuenensis*	AF008188	—	Fujian-China (Fujian province, China)	
14	*Paragonimus*	*macrorchis*	AF159598	AF159608	Thailand (Thailand)	
15	*Paragonimus*	*macrorchis*	KP784350	KP784357	Laos (Laos)	
16	*Paragonimus*	*microrchis*	MN656990	—	Yunnan-China (Yunnan province, China)	Sample31
17	*Paragonimus*	*microrchis*	—	—	Yunnan-China (Yunnan province, China)	Sample32
18	*Paragonimus*	*miyazakii*	AY618807	AY618757	Japan (Japan)	
19	*Paragonimus*	*miyazakii*	AY618834	AY618742	Fujian-China (Fujian province, China)	
20	*Paragonimus*	*ohirai*	U97214	U96911	Japan (Japan)	
21	*Paragonimus*	*proliferus*	AB663681	AB663678	Vietnam	
22	*Paragonimus*	*proliferus*	EU401809	EU401801	Yunnan-China (Yunnan province, China)	
23	*Paragonimus*	*proliferus*	EU401811	EU401803	Yunnan-China (Yunnan province, China)	
24	*Paragonimus*	*proliferus*	EU401812	EU401804	Yunnan-China (Yunnan province, China)	
25	*Paragonimus*	*proliferus*	MN656989	—	Yunnan-China (Yunnan province, China)	Sample30
26	*Paragonimus*	*sadoensis*	AF008190	—	—	
27	*Paragonimus*	*siamensis*	JQ322632	JQ322635	India (India)	
28	*Paragonimus*	*skrjabini*	AB703456	AB703448	Vietnam	
29	*Paragonimus*	*skrjabini*	AY618759	AY618729	Guangxi-China (Guangxi province, China)	
30	*Paragonimus*	*skrjabini*	AY618760	AY618743	Sichuan-China (Sichuan province, China)	
31	*Paragonimus*	*skrjabini*	AY618763	AY618748	Hubei-China (Hubei province, China)	
32	*Paragonimus*	*skrjabini*	AY618801	AY618730	Guangdong-China (Guangdong province, China)	
33	*Paragonimus*	*skrjabini*	AY618805	AY618734	Yunnan-China (Yunnan province, China)	
34	*Paragonimus*	*skrjabini*	MN656987	—	Yunnan-China (Yunnan province, China)	Sample28
35	*Paragonimus*	*westermani*	AB354223	AB354214	Thailand (Thailand)	

^*∗*^Sequences obtained in the study.

## Data Availability

The data used to support the findings of this study are available from the corresponding author upon request.
